# Supplementary material for: Predicting visual function by interpreting a neuronal wiring diagram
Source: Nature. 2024 Oct 2;634(8032):113–23. doi: 10.1038/s41586-024-07953-5 (PMC11446822; doi:10.1038/s41586-024-07953-5)
Supplement: Supplementary file 6 — CRF and ERF predictions for individual TmY4 and TmY9 cells. Analogous to Supplementary Data 3, but for TmY target types. Shown are the top four monosynaptic pathways, the strongest pathway passing through each of the top ten intermediary types (ranking from Extended Data Fig. 7), and the trisynaptic pathway Tm1–TmY–Dm3–TmY (see the section entitled Prediction of spatial normalization). [file 41586_2024_7953_MOESM6_ESM.zip › DataS4/TmY9q/720575940613029618.pdf]

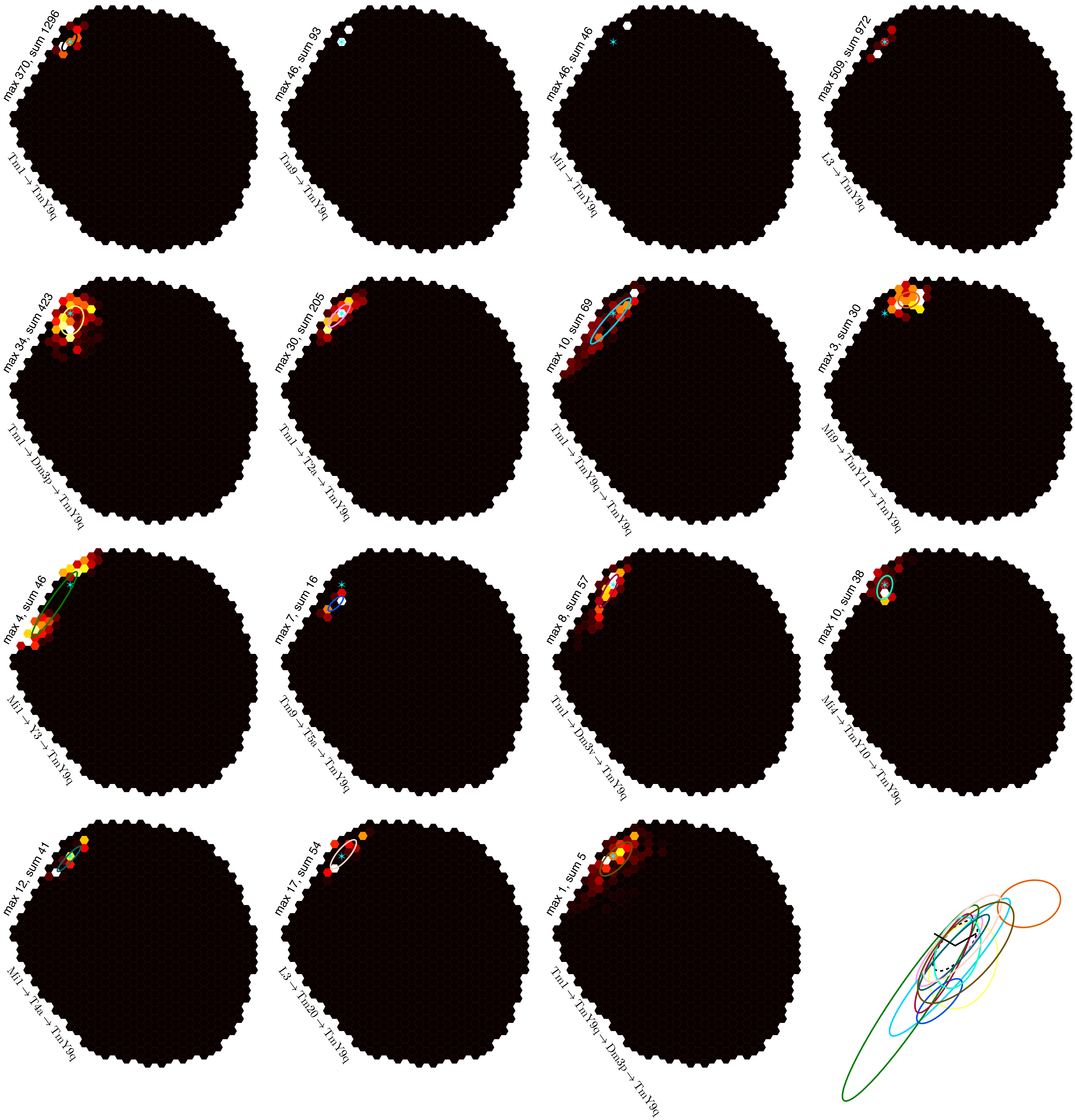
max 370, sum 1296
Tm1 → TmY9q
max 46, sum 93
Tm9 → TmY9q
max 46, sum 46
Mi1 → TmY9q
max 509, sum 972
L3 → TmY9q
max 34, sum 423
Tm1 → Dm3p → TmY9q
max 30, sum 205
Tm1 → T2a → TmY9q
max 10, sum 69
Tm1 → TmY9q → TmY9q
max 3, sum 30
Mi9 → TmY11 → TmY9q
max 4, sum 46
Mi1 → Y3 → TmY9q
max 7, sum 16
Tm9 → T5a → TmY9q
max 8, sum 57
Tm1 → Dm3v → TmY9q
max 10, sum 38
Mi4 → TmY10 → TmY9q
max 12, sum 41
Mi1 → T4a → TmY9q
max 17, sum 54
L3 → Tm20 → TmY9q
max 1, sum 5
Tm1 → TmY9q → Dm3p → TmY9q
